# Prevalência e Fatores Associados à SRAG por COVID-19 em Adultos e Idosos com Doença Cardiovascular Crônica: Uma Análise Crítica

**DOI:** 10.36660/abc.20210807

**Published:** 2022-04-07

**Authors:** Fernanda Gunha Ignácio, Fernanda Pinzon Ribeiro, Aline Oenning Baggio, Chaiana Esmeraldino Mendes Marcon

**Affiliations:** 1 Universidade do Sul de Santa Catarina Tubarão SC Brasil Universidade do Sul de Santa Catarina , Tubarão , SC – Brasil

**Keywords:** COVID-19, Coronavírus, Pandemia, Fatores de risco, Doença Cardiovascular

**Caro Editor,**

O estudo de Paiva et al. avaliou a incidência de pacientes infectados pelo vírus COVID-19, associado a doenças cardiovasculares (DCV), no Brasil. No estudo, conclui- se que a alta prevalência de síndrome respiratória aguda grave (SRAG) em adultos e idosos relaciona-se com características sociodemográficas, clínicas, sinais e sintomas. Visto isso, reitera-se a importância da atenção primária à saúde – a fim da manutenção das consultas periódicas, visando controle da doença e sintomatologia, ao passo que a presença de comorbidades cardiovasculares aumenta em casos severos de COVID-19. ^1^

No estudo, a população estudada foi de 116.343 pacientes, dos quais 61,9% foram diagnosticados por SRAG causada por COVID-19. Ao mesmo tempo, o estudo demonstra que a presença de doenças crônicas pode ser considerada fator de risco à infecção por COVID-19, devido a maiores vulnerabilidade e morbimortalidade. Sendo assim, pacientes com DCV prévia são mais predisponentes a desenvolverem quadros mais graves. Entretanto, no sexo feminino, observou-se uma menor prevalência de SRAG por COVID-19 devido ao fato de haver variação entre a resposta imunológica e a susceptibilidade a infecções virais entre os sexos, o que gera, assim, diferenças na gravidade e evolução da doença. ^1^

Em Wuhan, na China, uma metanálise com 46.248 pacientes infectados analisou as comorbidades mais prevalentes, estando a DCV (5±4%) em terceiro lugar. Foram avaliados, por Wang e colaboradores, 2020, somente pacientes hospitalizados acometidos pela infecção viral, os quais apresentaram maior prevalência – 19,6% – de DCV, um dado reforça o fato de que a comorbidade DCV contribui para o agravo da gravidade da COVID-19, visto a evidente necessidade de internação. Além disso, os pacientes demonstraram evolução com maior hipoxemia e imprescindível internação em UTIs. ^2^ O estudo realizado por Melo evidenciou resultados semelhantes a achados de um estudo na Itália – ambos analisados no decorrer de sete dias no mês de março de 2020; constatou-se decréscimo de 13% de pacientes com infarto agudo do miocárdio (IAM) quando associados na mesma semana do ano de 2019. Por outro lado, mesmo ocorrendo uma redução de casos de IAM e da taxa de números de óbitos hospitalares, houve um aumento na taxa de letalidade intra-hospitalar nas internações por DCV. Ambos os estudos demonstraram a relação da COVID-19 com a alta taxa de prevalência de lesões cardíacas e um grande potencial de gravidade da COVID-19 nas DCV, em que a letalidade em pacientes internados com DCV alcançou parcela mais economicamente ativa da população – de 20 a 59 anos. ^3^

Visto isso, destaca-se a importância do acompanhamento médico a pacientes portadores de doenças crônicas, já que, segundo Askin et al., no ano de 2020, houve aumento acentuado no dano do miocárdio de pacientes com COVID-19, aumentando o risco de morbimortalidade. Dessa forma, a valorização da DCV como complicação associada ao vírus COVID-19, devido ao agravamento da sintomatologia da doença, é de extrema significância para a atenção básica à saúde. ^4^

A presente situação exige estratégias que visem a prevenção de complicações associadas a doenças crônicas, como DCV. Portanto, os dados atuais demonstram a necessidade de atenção especial aos pacientes do grupo de risco assim como um manejo adequado das complicações cardiovasculares, visando uma rápida identificação e aplicação de tratamento adequado. Ademais, recomenda-se aos portadores de DCV a atualização das vacinas devido ao risco de infecção bacteriana secundária pelo SARS-CoV-2, assim como a adesão à dieta adequada, sono regular e atividade física, evitando o tabagismo e etilismo. ^2^

## Carta-resposta

Agradecemos aos autores pela análise submetida “Prevalência e Fatores Associados à SRAG por COVID-19 em Adultos e Idosos com Doença Cardiovascular Crônica: Uma Análise Crítica”, em especial pela exposição de dados que reforçam os achados apresentados em nosso estudo. ^1^ Destaca-se também a relevância da análise em relação ao aumento das taxas de letalidade intra-hospitalar nas internações por doenças cardiovasculares (DCV) como fator potencializador da necessidade de valorização deste grupo de risco na análise das complicações associadas à COVID-19. ^2 , 3^

As DCV já são estabelecidas como marcadores de risco na atenção primária à saúde (APS) brasileira, com o intuito de garantir a longitudinalidade do cuidado ao usuário e o desenvolvimento de estratégias preventivas voltadas ao cuidado integral da comunidade, ^4^ porém é essencial o questionamento quanto à aplicabilidade destas ações na estratégia de saúde da família (ESF). O desafio do monitoramento ao usuário como forma de gestão às DCV sugere ultrapassar o imperativo das ações que apoiam o sistema diagnóstico e terapêutico no cuidado e manejo às condições crônicas. ^5^ Neste sentido, um estudo publicado recentemente, com mais de 100 milhões de participantes de todo o mundo, destaca a importância da prevenção, da detecção e do controle na atenção primária, inclusive em países de baixa e média renda, com propostas de ações como uso de mensagens de texto para acompanhar os usuários com fatores de risco. ^6^

No contexto atual de enfrentamento à pandemia da COVID-19, esta discussão torna-se ainda mais relevante, pois, conforme reforçado pelos autores que realizaram a análise crítica, o agravamento da sintomatologia da COVID-19 coloca as DCV como um fator de risco importante às ações de rastreio, manejo e intervenção precoce.

Estudos sugerem a criação de propostas de modelos de cuidados às condições crônicas, como as DCV, envolvendo formação de grupos focais, que obtiveram resultados positivos na adesão da comunidade, melhora de indicadores clínicos durante o acompanhamento, adoção de prática de autocuidado e melhor análise das prioridades no planejamento em saúde. ^6^ De forma similar, a Organização Mundial de Saúde (OMS) lançou um guia de implementação para gestão de DCV na atenção primária, com propostas de triagem, avaliação e manejo de riscos neste nível de atenção e educação em saúde para profissionais quanto ao rastreio de fatores de risco, intervenções no estilo de vida e encaminhamentos necessários. ^7^ Especialmente acerca do manejo em usuários com DCV e sintomas persistentes pós-COVID-19, a complexidade clínica e psicossocial proporcionada pela coexistência dessas condições implica a necessidade de um cuidado multiprofissional e de longo prazo. Finalizamos, assim, agradecendo à possibilidade de reforçar a necessidade de se rever as ações na atenção primária à saúde, em especial em nosso atual contexto de saúde.
