# Supplementary material for: Prevalência e Fatores Associados à SRAG por COVID-19 em Adultos e Idosos com Doença Cardiovascular Crônica: Uma Análise Crítica
Source: Arq Bras Cardiol. 2022 Apr 7;118(4):787–8. [Article in Portuguese] doi: 10.36660/abc.20210807 (PMC9007019; doi:10.36660/abc.20210807)
Supplement: Translation

# Translation: Prevalence and Associated Factors of SARS caused by Covid-19 in Adults and Aged People with Chronic Cardiovascular Disease: A Critical Analysis

**Authors:** Fernanda Gunha Ignácio, Fernanda Pinzon Ribeiro, Aline Oenning Baggio, Chaiana Esmeraldino Mendes Marcon

**Affiliations:** 1 Brazil Universidade do Sul de Santa Catarina, Tubarão, SC – Brazil

**Keywords:** COVID-19, Coronavirus, Pandemic, Risk factors, Cardiovascular disease

**Dear Editor,**

The study by Paiva et al. evaluated the incidence of patients infected with the COVID-19 virus, associated with cardiovascular diseases (CVD), in Brazil. It is concluded that the high prevalence of severe acute respiratory syndrome (SARS) in adults and in the elderly is related to sociodemographic and clinical characteristics, signs and symptoms. In view of that, the importance of primary health care is reiterated – in order to maintain regular medical visits aiming at controlling the disease and symptoms, while the presence of cardiovascular comorbidities increases in severe COVID-19 cases.^1^

The study included 116,343 patients, of whom 61.9% were diagnosed with SARS caused by COVID-19. At the same time, the study demonstrates that the presence of chronic diseases can be considered a risk factor for infection by COVID-19 due to greater vulnerability and morbimortality. Therefore, patients with previous CVD are more likely to develop more severe conditions. However, in females, there was a lower prevalence of SARS by COVID-19 because there is a variation between the immune response and the susceptibility to viral infections between sexes, which generates differences in disease severity and evolution.^1^

In Wuhan, China, a meta-analysis with 46,248 infected patients analyzed the most prevalent comorbidities, with CVD (5±4%) in third place. Wang et al., 2020 evaluated only hospitalized patients affected by viral infection, which has shown a higher prevalence – 19.6% – CVD, which reinforces the fact that the comorbidity of CVD contributes an increased severity of COVID-19, given the evident need for hospitalization. In addition, the patients evolved with higher levels of hypoxemia and urgent hospitalization in ICUs.^2^In the study conducted by Melo, the results were

similar to those found in a study in Italy – both analyzed over seven days in March 2020; it found a decrease of 13% of patients with acute myocardial infarction (AMI) associated in the same week of 2019. On the other hand, even though there was a reduction in AMI cases and in the rate of hospital deaths, there was an increase in the in-hospital lethality rate in hospitalizations for CVD. Both studies demonstrated the relationship of COVID-19 with the high prevalence of cardiac lesions and a great potential for COVID-19 severity in CVD, in which mortality of hospitalized patients with CVD reached the most economically active portion of the population – from 20 to 59 years of age.^3^

In view of that, the importance of medical follow-up of patients with chronic diseases is highlighted, since, according to Askin et al., in 2020, there was a marked increase in myocardial damage in patients with COVID-19, increasing the risk of morbimortality. Therefore, the appreciation of CVD as a complication associated with the COVID-19 virus, due to the increase of the disease symptoms, is of extreme significance for primary health care.^4^

The current situation requires strategies aimed at preventing complications associated with chronic diseases, such as CVD. Therefore, current data demonstrate the need for special attention to patients at high risk as well as proper management of cardiovascular complications, aiming at quickly identifying and applying adequate treatment. Furthermore, it is recommended that patients with CVD get vaccinated – due to the risk of secondary bacterial infection by SARS-CoV-2 – and adopt a proper diet, regular sleep and physical activity, avoiding smoking and alcohol consumption.

## Reply

We would like to thank the authors for the analysis “Prevalence and Factors Associated with SARS caused by COVID-19 in Adults and Aged People with Chronic Cardiovascular Disease: A Critical Analysis”, especially due to the exposure of data reinforcing the findings presented in our study.^1^The analysis is also relevant because it includes information regarding the increase of in-hospital mortality rates in hospitalizations due to cardiovascular diseases (CVD) as a factor that further calls for the need to draw special attention to this group at risk in the analysis of complications associated with COVID-19.^2 , 3^

CVDs are established as risk markers in the Brazilian primary health care (PHC) in order to guarantee the longitudinality of user care and the development of preventive strategies aimed at the integral care of the community,^4^but it is essential to question the applicability of these actions in the family health strategy (FHS). The challenge of monitoring the users as a way of managing CVD suggests overcoming the imperative of actions that support the diagnostic and therapeutic system in the care and management of chronic conditions.^5^A recently published study with more than 100 million participants from around the world highlights the importance of prevention, detection and control in primary care, including in low- and middle-income countries, with proposals for actions that include sending text messages to check on users with risk factors.^6^

In the current context of facing the COVID-19 pandemic, this discussion is even more relevant because, as emphasized by the authors who conducted the critical analysis, the worsening of the symptoms of COVID-19 places CVDs as an important risk factor that affect screening, management and early intervention actions.

Studies suggest the creation of proposals for models of care for chronic conditions, such as CVD, involving focus groups with positive results in the community, improvement of clinical indicators during follow-up, adoption of self-care practices and better analysis of the priorities in health planning.^6^Similarly, the World Health Organization (WHO) released an implementation guide for CVD management in primary care, with proposals for risk screening, assessment and management at this level of health care and education for professionals regarding the screening of risk factors, lifestyle interventions and the required referrals.^7^Especially regarding the management of users with CVD and persistent symptoms after COVID-19, the clinical and psychosocial complexity provided by the coexistence of these conditions calls for long-term multidisciplinary care. To conclude, we would like to express our gratitude for the possibility of reinforcing the need to review primary health care actions, especially in our current health context.
